# On the Keyhole Hypothesis: High Mutual Information between Ear and Scalp EEG

**DOI:** 10.3389/fnhum.2017.00341

**Published:** 2017-06-30

**Authors:** Kaare B. Mikkelsen, Preben Kidmose, Lars K. Hansen

**Affiliations:** ^1^Department of Engineering, Aarhus UniversityAarhus, Denmark; ^2^Section for Cognitive System, Department of Applied Mathematics and Computer Science, Technical University of DenmarkKongens Lyngby, Denmark

**Keywords:** EEG, ear-EEG, prediction, mutual information, mobility

## Abstract

We propose and test the keyhole hypothesis—that measurements from low dimensional EEG, such as ear-EEG reflect a broadly distributed set of neural processes. We formulate the keyhole hypothesis in information theoretical terms. The experimental investigation is based on legacy data consisting of 10 subjects exposed to a battery of stimuli, including alpha-attenuation, auditory onset, and mismatch-negativity responses and a new medium-long EEG experiment involving data acquisition during 13 h. Linear models were estimated to lower bound the scalp-to-ear capacity, i.e., predicting ear-EEG data from simultaneously recorded scalp EEG. A cross-validation procedure was employed to ensure unbiased estimates. We present several pieces of evidence in support of the keyhole hypothesis: There is a high mutual information between data acquired at scalp electrodes and through the ear-EEG “keyhole,” furthermore we show that the view—represented as a linear mapping—is stable across both time and mental states. Specifically, we find that ear-EEG data can be predicted reliably from scalp EEG. We also address the reverse view, and demonstrate that large portions of the scalp EEG can be predicted from ear-EEG, with the highest predictability achieved in the temporal regions and when using ear-EEG electrodes with a common reference electrode.

## 1. Introduction

Neurotechnology will be a key component in future personalized services and health care. Many applications will be based on “mental state decoding,” i.e., the process of estimating the cognitive state of the human brain from quantitative measures. The main barriers to a broader application of mental state decoding in the “wild” are lack of mobility, comfort, and robust decoding schemes. Wearable EEG is a candidate for long term mental state monitoring (Casson et al., 2010; McDowell et al., 2013; Lin et al., 2014; Meyer et al., 2016). Among wearable platforms so-called ear-EEG is of special interest, since it enables recording of EEG in an unprecedented discreet and minimally intrusive way. Ear-EEG is recorded from electrodes placed in the outer ear, see Kidmose et al. (2013). As conventional EEG, ear-EEG reflects aggregated cortical activity as demonstrated in Mikkelsen et al. (2015). Given the spatial constraints of the ear, ear-EEG is typically based on a small number of electrodes, hence, providing for a relatively narrow field of view, which can be conceptualized as a *keyhole*. We will elaborate further on this metaphor in the discussion. The signals measured by ear-EEG have been characterized by core EEG experimental measures, such as event-related potential (ERP) responses to auditory and visual stimuli (Kidmose et al., 2013) and in terms of auditory steady state responses (ASSR) (Kidmose et al., 2012) for a recent discussion of the state of the art, see Mikkelsen et al. (2015). These results together indicate that when considering data at the aggregate level, i.e., averaging over multiple instances, the EEG signals propagating to the ear carry useful information about the brain processes within the keyhole's field of view.

In this work we aim to expand the analysis of the relation between the EEG signals measured in the ear and at the scalp. In particular, we analyse the sample-to-sample scalp-ear EEG relation from the point of view of the keyhole hypothesis, i.e., as a communication channel. We show that the Shannon communication capacity can be lower bounded by optimizing a linear predictive map between scalp and ear-EEG signals. We aim to quantify the channel capacity and its variability using machine learning methods, including a study of the very long term stability of the predictive relation between the input and the output of the communication channel.

## 2. Theory: mutual information and predictability

Considering instantaneous multivariate signals recorded from a set of scalp positions and multiple locations in the ear as random variables, we are interested in the ability of one set to predict the other, i.e., the possibility of a predictive relation between one set of measurements and a given electrode in the other set. For readability we first focus on the scalp to ear prediction problem.

For a given point in time let *x* be the vector of measured scalp signals and let *y* be the instantaneous signal recorded from an ear electrode at the same time. The mutual information between these two variables is defined as

(1) $$\begin{array}{r} I=\int p\left(x,y\right)\log\left(\frac{p\left(x,y\right)}{p\left(x\right)p\left(y\right)}\right)\;dydx\;, \end{array}$$

The functions *p*(*x*), *p*(*y*) represent the marginal probability density functions of the scalp and ear signals, while *p*(*x, y*) is the joint probability density of two signals. For further discussion and references on mutual information, see e.g., Paninski (2003).

**Figure 1:**
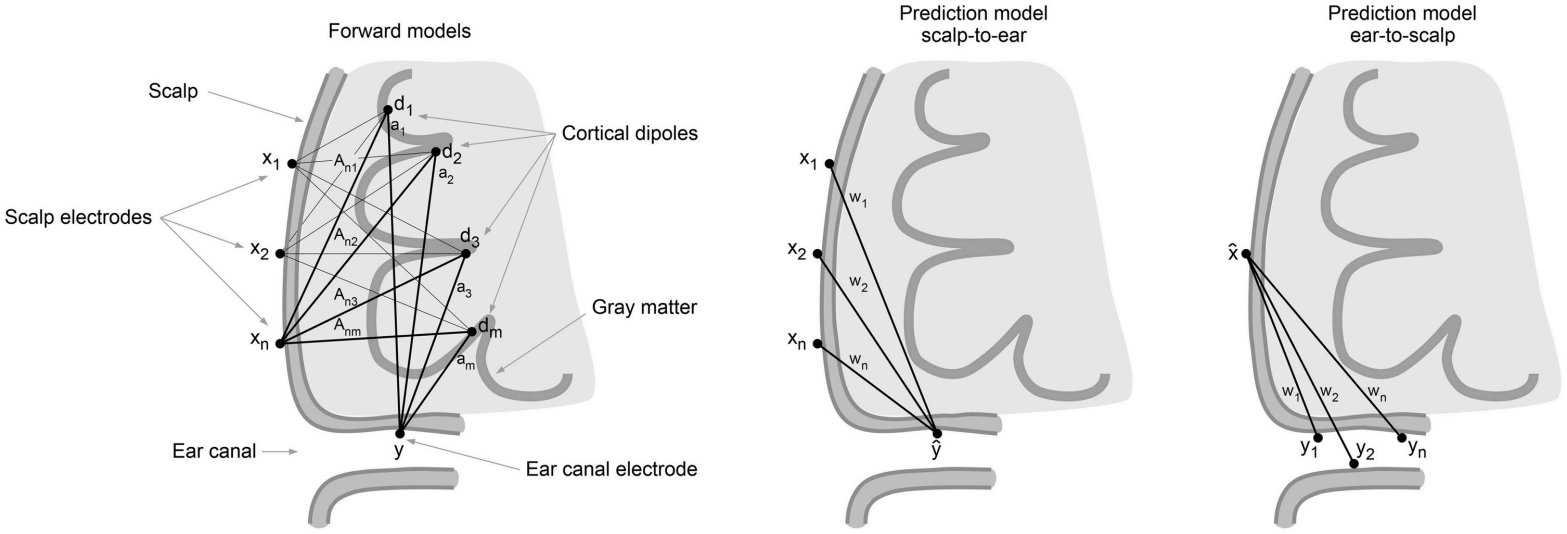
**Left**: Illustration of the forward models A,a from equivalent cortical dipoles d to scalp and ear canal electrodes x,y, respectively. **Center**: illustration of the predictive model w from scalp to ear. **Right**: Illustration of predictive model from ear to scalp.

Brain related EEG signals are well represented as synchronous activity in mesoscopic patches; so-called equivalent dipoles. The relations between scalp and ear-EEG measurements and the dipole is linear, quantified by the electrical forward models *A, a*, respectively,

(2) $$\begin{array}{r} x=Ad+\nu \end{array}$$

and

(3) $$\begin{array}{r} y=a^{\text{T}}d+\eta , \end{array}$$

where *A, a* represent the forward paths to the scalp and ear electrodes, while ν, η are non-brain signals. Together, (2) and (3) suggest a linear relation between dipole generated signals at the scalp and in the ear

(4) $$\begin{array}{r} y=w^{\text{T}}x+\epsilon , \end{array}$$

where ϵ are signals measured in the ear and not predictable by signals measured at the scalp, i.e., ear-specific non-brain signals and potential brain dipole signals invisible to the scalp electrodes. Such brain signals, denoted *d*_⊥_ would be orthogonal to all rows of the scalp forward model *A*, while non-orthogonal to the ear electrode forward model *a*. The massive spatial averaging effects of volume conduction (Holsheimer and Feenstra, 1977) will here come to assistance and reduce *d*_⊥_. The usefulness of the linear map will depend on multiple factors including head and tissue geometry, electrical conductivities, and the signal to noise characteristics of the relevant dipole signals generating the measured signals at the scalp and in the ear. An illustration of the forward model geometry is shown in Figure 1.

With Equation (4) and the data processing equation (Paninski, 2003), the mutual information between an ear electrode and the scalp electrodes may be lower bounded by the mutual information between *y* and its linear predictor $\hat{y}\left(x\right)=w^{\text{T}}x$,

(5) $$\begin{array}{r} I\geq I_{\hat{y},y}=\int p\left(\hat{y},y\right)\log\left(\frac{p\left(\hat{y},y\right)}{p\left(\hat{y}\right)p\left(y\right)}\right)\;d\hat{y}dy. \end{array}$$

Within a joint multivariate normal approximation of two signals distributions, the mutual information can be written simply in terms of their correlation coefficient, ρ, see e.g., Granger and Lin (1994)

(6) $$\begin{array}{r} I_{\hat{y},y}=\int p\left(\hat{y},y\right)\log\left(\frac{p\left(\hat{y},y\right)}{p\left(\hat{y}\right)p\left(y\right)}\right)\;d\hat{y}dy=-\frac{1}{2}\log\left(1-\rho^{2}\left(\hat{y},y\right)\right). \end{array}$$

Considering the link between scalp and ear as a communication channel, the Shannon capacity (Cover and Thomas, 2012) is given as

(7) $$\begin{array}{rcl} C & = & \underset{p\left(\hat{y}\right)}{\text{max}}\int p\left(\hat{y},y\right)\log\left(\frac{p\left(\hat{y},y\right)}{p\left(\hat{y}\right)p\left(y\right)}\right)\;d\hat{y}dy\\ =\underset{p\left(\hat{y}\right)}{\text{max}}\left(-\frac{1}{2}\log\left(1-\rho^{2}\left(\hat{y},y\right)\right)\right). \end{array}$$

With respect to the weights of the linear predictor, $\hat{y}=w^{\text{T}}x$, the maximum correlation coefficient is obtained when

(8) $$\begin{array}{r} w\propto \Sigma_{x,x}^{-1}\Sigma_{x,y} \end{array}$$

i.e., proportional to the least squares estimate, where $\Sigma_{x,x}=E\left(xx^{\text{T}}\right)$ and Σ_*x, y*_ = 𝔼(*xy*). We note that estimating the mutual information viz. the correlation coefficient on the same set as we estimate the weights will in general lead to an unknown bias. To obtain an unbiased estimator we estimate the weights of the linear map on a training set and evaluate the correlation coefficient on separate test sets. This furthermore allows us to measure and illustrate possible non-stationarities of the estimator and predictions.

## 3. Data

In this paper, data from two different experiments is used to evaluate the mutual information and capacity of the scalp and ear-EEG communication channel. In both cases, during recording, scalp EEG and ear-EEG shared a reference (*C*_*z*_). This link was subsequently removed by preprocessing such that scalp data and each ear (three groups in all) were referenced to their respective averages. This re-referencing simulates a situation in which the signal groups are independently acquired. Further details of the ear-EEG setup can be found in Mikkelsen et al. (2015).

The two experiments are:

*Laboratory dataset:* Ten subjects aged 23–43 (median 30) were subjected to a battery of EEG-related paradigms, as described in Mikkelsen et al. (2015). Of importance to this study, the paradigms included an alpha-attenuation task and an ERP-task. The alpha-attenuation task required the participant to alternate between open and closed eyes, while performing a simple arithmetic task, and the ERP-task consisted of a classical auditory odd-ball paradigm, adapted from Näätänen et al. (2004). The 32 scalp channels were C1-6, CP3-6, F7-8, FC3-6, FCz, FT7-8, Fz, P3-8, T7-8, and TP7-10. The sampling rate was 256 Hz. Scalp electrodes were embedded in a cap. Each ear had six electrodes, as described in Mikkelsen et al. (2015).
*In the wild longitudinal dataset:* A single subject (aged 44) wore combined ear- and scalp EEG for a total of 12 h and 55 min. The subject performed normal life activities during the measurement. The 24 scalp channels were C3-4, CP1-2, CP5-6, Cz, F3-4, F7-8, FC1-2, FC5-6, Fz, M1-2, P3-4, P7-8, and T7-8. Data was originally sampled at 2,000 Hz, but was down sampled to 256 Hz prior to processing. Scalp electrodes were embedded in a cap. Each ear had four electrodes, ExI and ExE (inside the canal), and ExA and ExB (in the concha), see Kidmose et al. (2013).

The experiment was approved by the regional scientific ethics committee and by the national Danish Health and Medicines Authority^1^. As per the guidelines of these authorities, all participants were given written and oral information. All gave written, informed consent before participating, and after having had ample time for consideration.

## 4. Methods

### 4.1. Preprocessing

All data was filtered with a pass-band of 0.5–45 Hz, using EEGLAB (Delorme and Makeig, 2004). Additionally, in the case of the laboratory dataset, the first 6 s of each time series were discarded. As described in Mikkelsen et al. (2015), faulty ear-EEG channels were identified and discarded (in both data sets).

### 4.2. Model estimation and analysis

The weights of the predictive model were estimated using Equation (8) with expectations computed as empirical averages over cleaned training sets. Least squares estimation is sensitive to outliers (Rousseeuw, 1984), and outliers can indeed be abundant in EEG caused by electro-physiological artifacts, such as blinks and muscle activity, by electrode motion and multiple other external influences. Since we are not limited by data in the present context we aimed at identifying outliers by aggressive outlier rejection (Markou and Singh, 2003). In particular we estimated the sample covariance matrices of both scalp and ear-EEG, computed the Mahalanobis distance of individual samples to the sample median and rejected the 15% highest distances within each measure separately, safely removing outliers in both scalp and ear signals according to visual inspection.

The model was estimated separately for each subject. The correlation coefficient, ρ, was calculated using the corrcoef.m command in Matlab^2^.

## 5. Results

**Figure 2:**
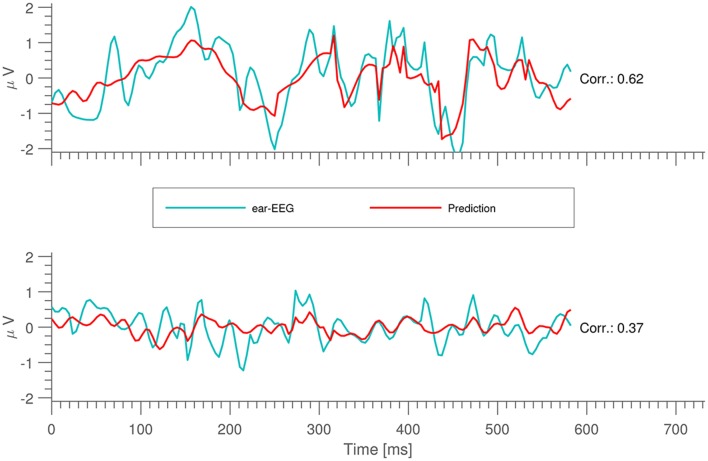
Short snippets of ear-EEG measurements together with their predictions.

**Figure 3:**
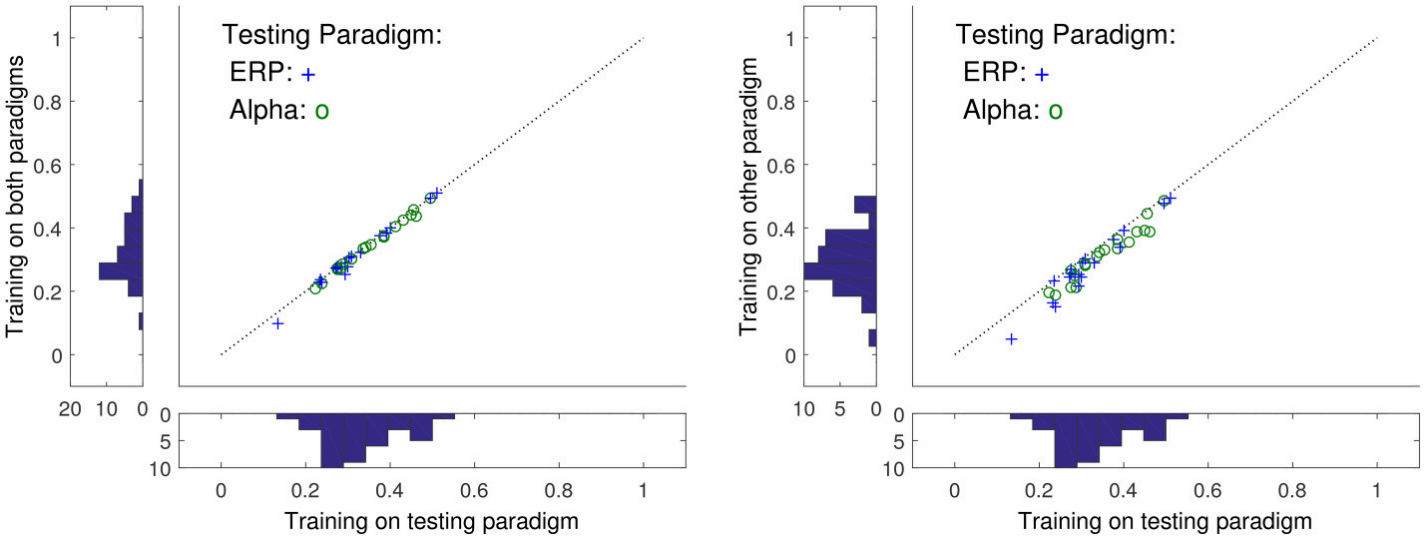
The relation between ρ when the training and test sets are varied. For the x-axes are reported ρ when test and training sets come from the same task (ERP or alpha paradigms), while the values on the y-axes correspond to training data drawn from a different task. Histograms are marginal distributions. **Left**: Training data was here 2 min of alpha and ERP tasks (4 min in total). **Right**: Training was here 2 min of alpha/ERP for ERP/alpha test data.

The performance of the prediction models was evaluated as the correlation between the measured and the predicted signals on the test sets. Figure 2 shows examples of short time segments of measured and predicted ear-EEG signals for cases with both high and low cross correlation. More generally, the distribution of prediction-EEG correlations is displayed in Figure 3. A spread in correlations is found ranging from ~0.1 to ~0.6.

### 5.1. Stability, generality and uniqueness

Also seen in Figure 3 is the resulting ρ's as the training and test stimulus paradigms are interchanged. While changing the type of data used in testing generally reduces ρ, the correlation is mostly conserved if the training data contains measurements from several paradigms including the one of the test data. This suggest that a sufficiently general training data set could generate a general predictor valid for all stimuli type scalp data.

**Figure 4:**
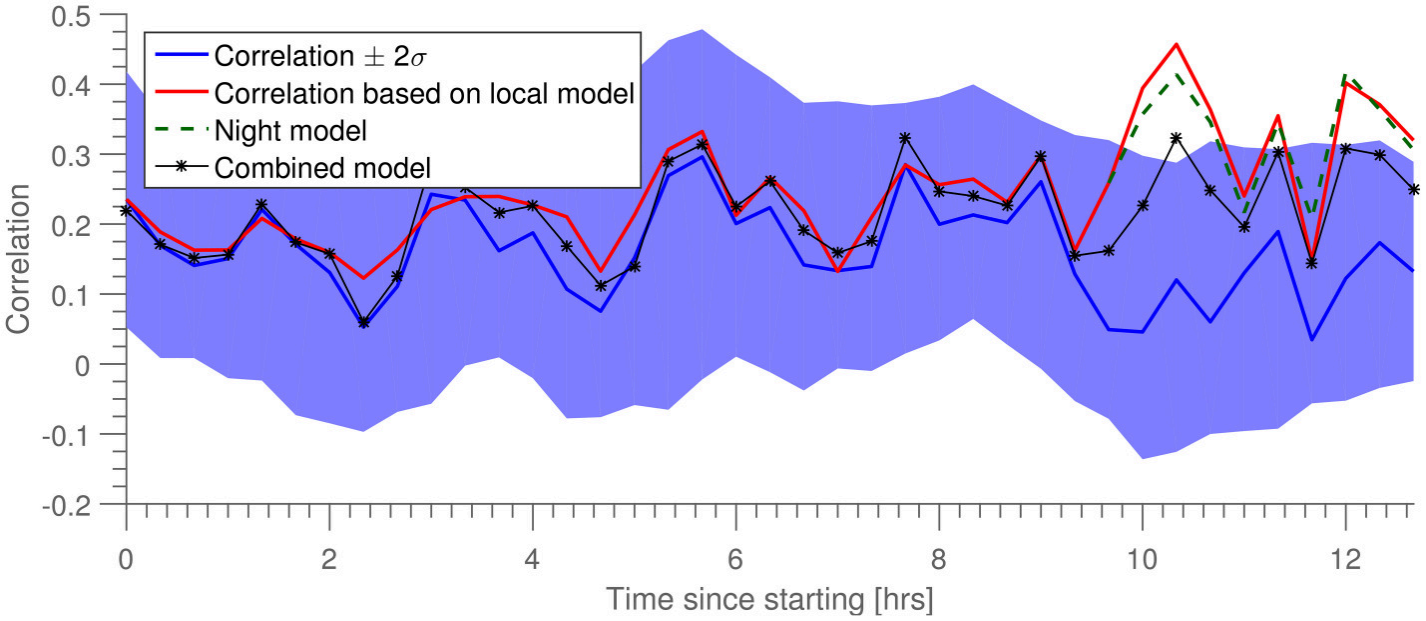
Prediction correlation, ρ, as a function of time since training (in blue). Shown in red is prediction correlation for a model trained on data within the last 10 min. “Night model” is a prediction based on the red model from the time when the subject went to sleep. “Combined model” is a prediction based on combining the night training data with data from the beginning of the dataset.

An additional stability metric is the behavior of the prediction quality as a function of time. In Figure 4 is shown the correlation, ρ(*t*), for both a fixed predictor trained on the first 2 min of data (blue line), and a “local” predictor, described in the following. The predictions were obtained by stepping through the longitudinal 13-h data set with a time step of 20 min. After each step, 2 min of data is used to train a “local” model, while the following 8 min were partitioned into 3-s intervals on which both local and original models were used. From the distribution of correlation ρ for each of these sets of 3-s intervals, we estimated mean and standard deviation of ρ. In Figure 4 we show both these estimates for the original model (in blue), as well as the estimated mean ρ for the local model (in red).

While the correlation for the prediction by the local model is generally higher than that of the long term—“global”—model, they do exhibit similar temporal trajectories. We hypothesize that the variability of prediction quality (particulary the points of where prediction is challenged) is driven by a combination of artifacts (non-brain signals) and general non-stationarity of the brain signals. Toward the end of the measurement we see a clear departure between “global” and “local” predictors. This point of departure happens to coincide quite well with the subject going to bed, and we hypothesize that the decay of the prediction quality at this point is again due to the non-stationarity induced by sleep, since the long term model (blue) was trained on wake data and thus the predictive performance is lower on sleep data. To test this hypothesis, we introduced a third model (“Night model,” in green) trained on data immediately after the subject went to sleep. We see that this model continues to perform well for the remainder of the data set. We postulate that if the measurement had continued until morning, the “local” and “global” lines would have converged again, and “Night Model” would have dropped below “global.” As a further test, we introduced a fourth model, trained on both the training set for the “global” model as well as the “night” model (“Combined model”). We see that this achieves equal prediction quality during both night and day portions of the data.

It is interesting to see the small, yet general, increase in prediction quality during sleep, c.f., the recent results presented in Zibrandtsen et al. (2016) that gave an example of ear-EEG's ability to capture relevant sleep stages as identified by scalp EEG.

**Figure 5:**
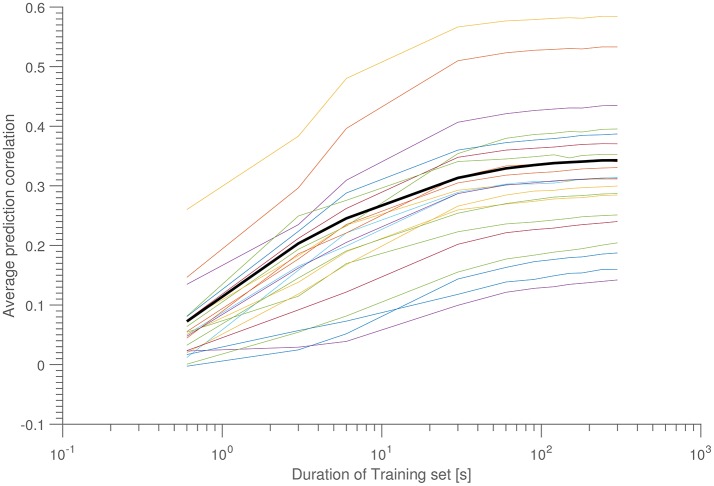
Average correlation as a function of training set size. Each colored line corresponds to a 15-min data set, while the bold black line is the population average. Each data points consists of the average over 10 sets of 9 test data. All data was taken from the ERP-part of the “laboratory data set.”

As a final stability test, Figure 5 shows ρ as a function of amount of training data. In this scenario, 10 time intervals of equal length were scattered throughout a 15-min interval (corresponding to a measurement from the “Laboratory” dataset), and each interval in turn was used as a training data set, which was tested on the other 9 intervals (so 90 tests were performed in total, for each 15-min data set). For each interval length is reported the mean of these 90 tests. Each line corresponds to a different 15-min data measurement (hence, belonging to different subjects).

### 5.2. Prediction of event-related potentials

**Figure 6:**
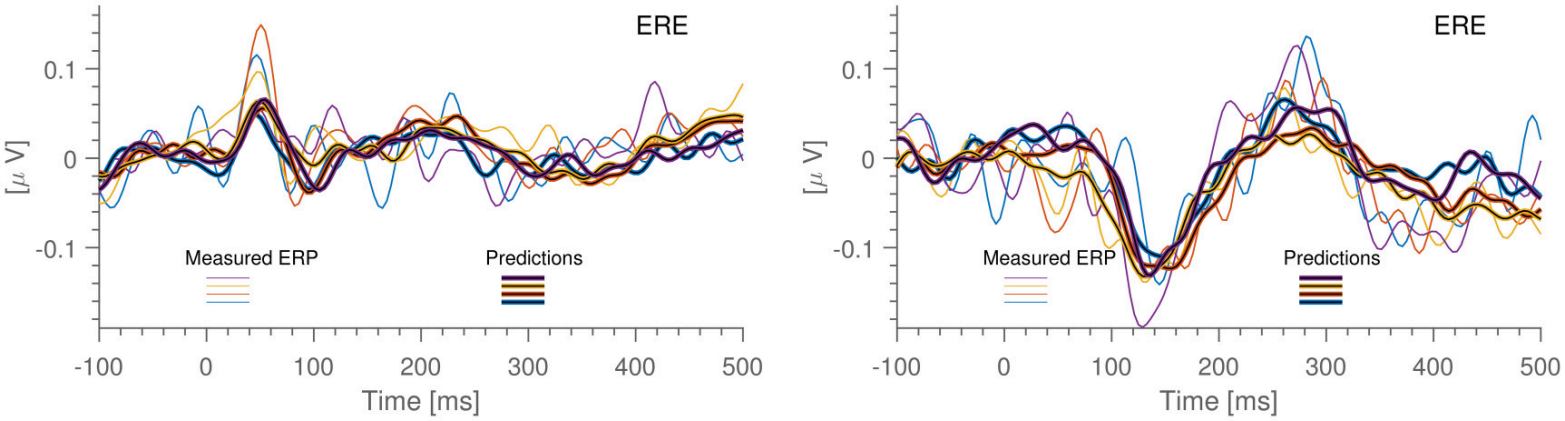
Result of running scalp-based ERPs through the prediction algorithm (blackened) together with ERPs calculated from the ear-EEG data. Each color corresponds to 15 min of measurements. All data is from the same individual, electrode ERE. The ERPs are the so-called difference waves, obtained by deducting the ERP of the standard “beep” from the ERP of the pooled “oddball beep”. **Left**: Auditory ERP. **Right**: Mis-Match Negativity ERP (MMN).

To investigate to which extent the predicted signals reflect cortical activity, we estimated the ERP from both the measured and the predicted ear signals. Specifically, the ERP was estimated from standard stimuli epochs from the auditory mismatch negativity (MMN) paradigm in the Laboratory dataset. In Figure 6 (left) is shown the auditory onset response from one of the subjects; we observe a good correspondence between the measured and predicted ERP waveforms and the timings of the components are close to what have been previously reported (Picton et al., 1974). In Figure 6 (right) is shown the so-called MMN response, which is the difference between the ERP estimated from standard and oddball stimuli. This particular subject had a clear 150 ms component in both the measured and the predicted MMN response. This suggests that while (Mikkelsen et al., 2015) did not find a significant MMN response in the ear-EEG across the 13 subjects, it may be possible in a subset of individuals. Additionally, the fact that predicted and measured ERPs are seen to be in excellent correspondence is further evidence that the high correlations reported above are not caused by confounding signals appearing in both datasets. Finally, we note that the predicted ERP has a significantly lower noise level. This is most likely due to the fact that the prediction inherits the higher SNR of the scalp data as well as the fact that the prediction algorithm combines data from multiple EEG channels, causing the individual scalp electrode noise components to average out.

### 5.3. Predicting scalp from ear data

**Figure 7:**
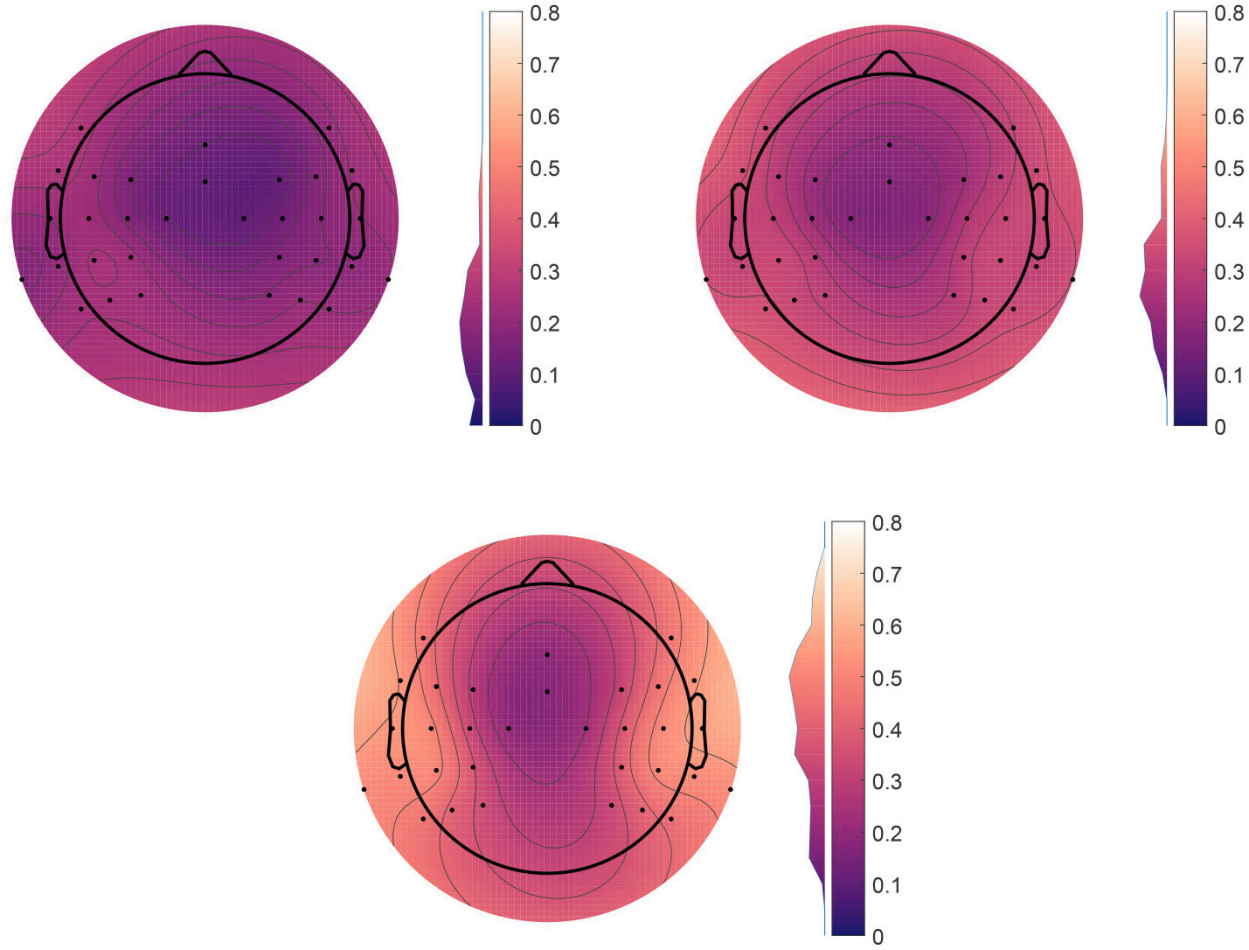
Scalp plots of prediction correlation for each scalp channel, as predicted from ear-EEG data. We see that in the regions close to the ears, the mutual information is high. Each data point is the average correlation over both α and ERP paradigms, averaged over all 10 subjects. The histogram next to the color bar shows the distributions of the correlations before averaging. **Top right**: Using only the left ear. **Top left**: Decoupled ears. **Bottom**: Coupled ears, with common reference.

In the above, we have focused on the communication channel from the point of view of ear-EEG. However, as illustrated in Figure 1 we can expect the scalp-ear channel to be bidirectional. We thus explore the reverse mappings letting *y* be a scalp electrode and let x represent either of the two sets of four ear-EEG electrodes (left or right) or the eight electrodes combined (i.e., with common reference). In Figure 7 is shown test correlations for the scalp data predicted from ear data. Data is shown for the three different scenarios mentioned. The overall prediction quality increases as more ear-EEG information is made available. While the correlation drops below 0.1 for the central scalp electrodes when prediction is based on a single ear, the correlation values are high in the temporal regions, reaching >0.6 when both ears are used for the prediction and have common reference.

## 6. Discussion and conclusions

Due to volume conduction, EEG signals from all locations in the brain are distributed widely in the human head. This opens the possibility that electrodes in unconventional positions, such as the ear, may be used to pick up signals from distant locations. In technical terms: A large fraction of the signal of an EEG electrode can be predicted from the remaining EEG electrodes. While high density EEG has been proven to open doors to numerous mental processes, we may think of the few electrode EEG as in the case of ear-EEG giving a keyhole's view. The objective of the theoretical framework and the analysis of EEG signals presented in this paper was to substantiate this statement.

Specifically, we aimed at investigating how the information observed through the keyhole relates to cortical processes, and how stable the projection of these cortical processes are over time and type of mental state. A long term aim of this research is to decode mental states based on low dimensional EEG, specifically EEG acquired from wearable devices as e.g., embodied by the ear-EEG platform. Therefore, we have used data recorded from conventional scalp EEG and from electrodes placed in the ear. To further test the keyhole hypothesis EEG measured from ear electrodes were compared to EEG predicted from the scalp EEG.

First, to evaluate the generality of the predictor, it was investigated how well a predictor trained on one dataset performed in prediction of another dataset. For that purpose EEG recordings from two fundamentally different paradigms were used: an exogenous paradigm where the origin of the responses is in the auditory pathway and primary auditory cortex located in the temporal lobes, and an endogenous paradigm where the responses primarily originate from the occipital and parietal lobes. Even though these datasets represent very different cortical activations, only minor impact on the prediction performance was observed, see Figure 3. We conjecture that this generality is achieved because the scalp EEG largely represents aggregated activity from the whole cortex and therefore the paradigm specific activation has only a minor effect. Second, the stability of the predictor over time was studied based on a 13-h recording, in which the subject went through an uncontrolled variety of states. The prediction performance remained largely stable over the time course, demonstrating that the prediction model was stable across both time and states, see Figure 4. Third, estimates of ERPs from both the predicted and measured EEG revealed a high degree of similarity, see Figure 6, demonstrating that the predicted EEG conveyed cortical information. Finally, and possibly most importantly, we show in Figure 7 that not only is the information within ear-EEG to a large extent contained within the scalp-EEG; a respectable portion of the scalp-EEG can also be reconstructed from ear-EEG. We believe that the results presented in this paper provide evidence in support of the keyhole hypothesis and highlight the future perspectives of ear-EEG, especially the possibilities regarding development of combined paradigms in which ear-EEG data is combined with prior knowledge gained through more traditional methods with greater spatial resolution.

We note that both the linear map itself and the quality of predictions depend on multiple factors and thus is to be quantified experimentally. The geometry of the head plays an important role, e.g., the proximity of natural holes in the skull which may influence the result as discussed in Torre et al. (1999) and Heasman et al. (2002).

In summary there is generally a high mutual information between signal monitored at the scalp and in ear-EEG. The high correlations observed between predictions and measurements (Figure 1) are remarkably stable within several metrics (Figures 3–5).

Our objectives and methods are related to a number of previous works. Investigations of wearable EEG are already numerous, see e.g., Lin et al. (2009), Juhl et al. (2010), Chi et al. (2013), Stopczynski et al. (2014), Debener et al. (2015), and Wang et al. (2015), for studies discussing mobile EEG and demonstrating the detection of signals with portable, wearable and keyhole like EEG devices. The observation that volume conduction leads to wide distribution of signals is often used to interpolate the spatial EEG signal after removal of individual electrodes, see e.g., Junghöfer et al. (2000). A similar idea is used to propose a denoising mechanism for scalp EEG signals in De Cheveigné and Simon (2008b) and for removal of artifacts by the same authors in De Cheveigné and Simon (2008a).

Finally, returning to the keyhole hypothesis: Ear-EEG offers poorer spatial resolution of the underlying processes compared to scalp EEG, but the high mutual information between scalp and ear channels indicate that the time courses of a broad set of processes—characterized using scalp EEG under lab conditions—may later be detected “in the wild” using ear-EEG. Thus, we believe that the results presented in this paper highlight the future possibilities of ear-EEG, especially the possibilities regarding development of combined paradigms in which ear-EEG data is used together with prior knowledge gained through traditional brain scanning modalities. One example of this, as hinted at in this work, is to use scalp-based predictions when planning ear-EEG experiments, viz. Figure 6. This could reveal ahead of time whether results would be likely to be obtained in a full-scale experiment. One thing to keep in mind in this regard, however, is that we have found that the predictive models are sensitive to the placement of the scalp electrodes. Thus, a model trained on measurements from 1 day may not be accurate when used to predict ear-EEG data based on scalp measurements the next day, since the cap placement could likely be different.

## Author contributions

The core idea of the work was contributed by LH and PK. Theory was developed by LH. Measurements was performed by KM and PK. Data analysis was conducted by LH and KM and they wrote the paper.

### Conflict of interest statement

The authors declare that the research was conducted in the absence of any commercial or financial relationships that could be construed as a potential conflict of interest.

## Footnotes

^1^Danish: “De Videnskabsetiske Komiter for Region Midtjylland,” case no: 1-10-72-48-15; and Danish: “Sundhedsstyrelsen,” case no: 2014080169.

^2^Mathworks https://www.mathworks.com/products/matlab.html
